# Study of the Association between microRNA (miR-25T>C, miR-32C>A, miR-125C>T, and miR-222G>T) Polymorphisms and the Risk of Recurrent Pregnancy Loss in Korean Women

**DOI:** 10.3390/genes11040354

**Published:** 2020-03-26

**Authors:** Jeong Yong Lee, Jung Oh Kim, Han Sung Park, Chang Soo Ryu, Ji Hyang Kim, Young Ran Kim, Woo Sik Lee, Jung Ryeol Lee, Nam Keun Kim

**Affiliations:** 1 Department of Biomedical Science, College of Life Science, CHA University, Seongnam 13488, Korea; smilee3625@naver.com (J.Y.L.); jokim8505@gmail.com (J.O.K.); hahnsung@naver.com (H.S.P.); regis2040@nate.com (C.S.R.); 2 Department of Obstetrics and Gynecology, CHA Bundang Medical Center, School of Medicine, CHA University, Seongnam 13496, Korea; bin0902@chamc.co.kr (J.H.K.); happyimam@naver.com (Y.R.K.); 3 Department of Obstetrics and Gynecology, CHA Gangnam Medical Center, School of Medicine, CHA University, Seoul 06135, Korea; wooslee@cha.ac.kr; 4 Department of Obstetrics and Gynecology, Seoul National University Bundang Hospital, Seongnam 13590, Korea

**Keywords:** pregnancy, variant, mutation, DNA, gene, microRNA

## Abstract

Recurrent pregnancy loss (RPL), which is defined as two pregnancy losses that occur before 20 weeks of gestation, is relatively common, occurring in approximately 1–5% of women. The underlying cause is often unclear, although numerous factors may contribute to RPL, including environmental and immunological factors, blood coagulation disorders, and genetics. In particular, single nucleotide variants have been associated with RPL, including those found in microRNAs (miRNAs). We investigated the association between four miRNA polymorphisms, miR-25T>C, miR-32C>A, miR-125aC>T, and miR-222G>T, and RPL in a cohort consisting of 361 RPL patients and 272 controls. Subjects were genotyped at miRNA loci by polymerase chain reaction-restriction fragment length polymorphism (PCR-RFLP) analysis, and genotype frequencies were calculated. We then performed allele and genotype combination analyses and measured the association between miRNA polymorphisms and clinical variables in both RPL patients and controls. We detected a statistically significant association between RPL and the miR-25T/miR-32C/miR-125aT/miR-222T allele combination (adjusted odds ratio (AOR), 4.361; 95% confidence interval (CI), 1.496–12.72; *P* = 0.003). Three-gene combinations, including miR-32C/miR-125aT/miR-222T (AOR, 3.085; 95% CI, 1.254–7.588; *P* = 0.010) and miR-25T/miR-125aT/miR-222T (AOR, 2.929; 95% CI, 1.183–7.257; *P =* 0.015), and the two-gene combination miR-125aT/miR-222T (AOR, 2.417; 95% CI, 1.084–5.386; *P =* 0.026) were also associated with RPL. Analysis of variance (ANOVA) revealed that platelet counts and blood urea nitrogen levels were significantly different in RPL patients expressing different miR-125aC>T and miR-25T>C genotypes, respectively (*P* < 0.05). In addition, creatinine levels were lower in RPL patients expressing the minor alleles miR-25T>C and miR-32C>A. We investigated miRNAs (miR-25, miR-32, miR-125a, miR-222) in RPL patients and healthy controls. Significantly different allele frequencies were detected by ANOVA. We suggest that miRNAs and clinical factors can impact RPL occurrence.

## 1. Introduction

Recurrent pregnancy loss (RPL), which is defined as at least two pregnancy losses (PLs) that occur before 20 weeks of gestational age [1], is a relatively common condition [2] that occurs in approximately 1–5% of reproductive-aged women [1]. Although the underlying cause of RPL is often unknown, a number of biological and environmental factors have been associated with the increased incidence of RPL [3], including genetic variants [4,5].

In particular, polymorphisms in microRNAs (miRNAs) have been linked to RPL. These small, non-coding RNAs are approximately 20–24 nucleotides (nt) in length and are involved in the regulation of many critical biological processes. Specifically, miRNAs bind in a sequence-dependent manner to the 3’-untranslated region (3’-UTR) of their target genes [6] and function to downregulate the gene expression through RNA silencing mechanisms, including translational inhibition and mRNA degradation [7]. Notably, single nucleotide polymorphisms (SNPs) have previously been reported to affect RPL occurrence [8]. miRNA SNPs, such as those reported for miR-423 and miR-125a, have also been associated with RPL occurrence in Chinese women [9]. In addition, in our previous studies, we found that polymorphisms in miR-150, miR-1179, miR-27a, and miR-449b were associated with an increased risk of RPL [10].

Maternal vascular development during pregnancy is very important [11] because both angiogenesis and vasculogenesis play key roles during placental formation. Accordingly, high levels of vascular endothelial growth factor (VEGF) and kinase insert domain receptor (KDR), also known as VEGF receptor 2, are required during early pregnancy and are necessary to maintain the condition of the uterus and placenta throughout fetal development [12]. VEGF induces cell migration and proliferation and is a key modulator of angiogenesis. KDR is one of the numerous VEGF receptors that are known to play important roles in angiogenesis in the fetal environment [13].

The cytokine transforming growth factor-β (TGF-β) has three isoforms [14], one of which functions as a positive regulator of angiogenesis [15]. TGF-β can also affect the differentiation of T helper (Th17) and regulatory T (Treg) cells. In the endometrium, a large quantity of Treg cells is necessary for successful implantation [16]. Another study reported that the transfer of Treg cells can protect the fetus in abortion-prone mice [17]. In a previous study, we found that miR-25, miR-32, miR-125, and miR-222 were associated with the TGF-β signaling pathway [18]. Because TGF-β superfamily members play crucial roles in female reproductive function and angiogenesis [18], we hypothesize that these miRNAs are also involved in reproductive system development and/or function. Accordingly, upregulated miR-32 levels have been found to promote preeclampsia, and miR-125a overexpression can result in abnormal pregnancies [19,20]. In addition, as noted above, SNPs in mir-125a have been linked to RPL.

To further determine whether miR-25, miR-32, miR-125, and miR-222 are associated with RPL, in this study, we selected four miRNA polymorphisms, miR-25 rs1527423 T>C, miR-32 rs7041716 C>A, miR-125a rs12976445 C>T, and miR-222 rs34678647 G>T, and measured their frequencies in Korean women with RPL and healthy controls. 

## 2. Materials and Methods

### 2.1. Study Population

We enrolled a total of 669 female participants, including 388 patients and 281 healthy controls. Participants were excluded randomly to perform age matching between groups; therefore, our final analysis included 633 samples from 361 female RPL patients and 272 female healthy controls. RPL was diagnosed and confirmed based on human chorionic gonadotropin (hCG) levels, ultrasonography, and physical examinations, prior to 20 weeks of gestation. RPL patients were enrolled in this study at the CHA Bundang Medical Center Infertility Medical Center (Seongnam, South Korea), from March 1999 to February 2012. Control participants were recruited from the CHA Bundang Medical Center and were confirmed to have a normal 46 XX karyotype, regular menstrual cycles, a history of at least one naturally conceived pregnancy, and no history of pregnancy loss [21]. None of the participants had a past history of smoking or alcohol use. 

Patients with pregnancy losses caused by anatomic, hormonal, autoimmune, or thrombotic factors were excluded from the study group. Anatomic abnormalities in RPL patients were identified by hysterosalpingogram, sonography, computed tomography, hysteroscopy, and magnetic resonance imaging (MRI). Altered hormone levels can indicate luteal insufficiency, hyperprolactinemia, or thyroid disease. To exclude subjects with these conditions, we measured the levels of thyroid-stimulating hormone (TSH), prolactin (PRL), follicle-stimulating hormone (FSH), free T4, luteinizing hormone (LH), and progesterone in peripheral blood. To exclude lupus and antiphospholipid syndrome as autoimmune causes of RPL, lupus anticoagulant and anticardiolipin antibodies were measured, as described in a previous study [22]. This study was approved by the Institutional Review Board of CHA Bundang Medical Center (IRB number: BD2010-123D, 21 June 2011), and written informed consent was provided by all patients.

### 2.2. Genotype Analysis

Genomic DNA was extracted from whole blood using the G DEX II Genomic DNA Extraction Kit (Intron Biotechnology Inc., Seongnam, Korea). DNA was diluted to 100 ng/µl with 1× Tris ethylene diamine tetra acetic acid (TE) buffer and, subsequently, 1 µl of each sample was used to amplify miRNA polymorphisms. Genotyping analysis was performed, as reported previously, using polymerase chain reaction-restriction fragment length polymorphism (PCR-RFLP) assays [23]; primer sequences for the amplification of each genotype and the experimental conditions used are identical to those reported in our previous study [18].

### 2.3. Assessment of Blood Coagulation Status

Blood samples were collected from RPL patients and control subjects during pregnancy. Platelets (PLT) were measured using the Sysmex XE 2100 Automated Hematology System (Sysmex Corporation, Kobe, Japan). Prothrombin time (PT) and activated partial thromboplastin time (aPTT) were measured with the ACL TOP automated photo-optical coagulometer (Mitsubishi Chemical Medience, Tokyo, Japan).

### 2.4. Assessment of Homocysteine, Folate, Total Cholesterol, and Urate Concentrations 

Plasma homocysteine, folate, total cholesterol, and urate concentrations, as well as blood coagulation factors, were measured after fasting for 12 h. Homocysteine levels were measured using a fluorescence polarization immunoassay and the Abbott IMx Analyzer (Abbott Laboratories, Abbott Park, IL, USA). Folate levels were determined using a competitive immunoassay with ACS:180 (Bayer Diagnostics, Tarrytown, NY, USA). Total cholesterol and urate levels were determined using commercially available enzymatic colorimetric tests (Roche Diagnostics, Mannheim, Germany). 

### 2.5. Statistical Analysis

Genotype and haplotype frequencies in RPL and control subjects were compared using multivariate logistic regression. Allelic frequencies have been calculated based on Hardy Weinberg equilibrium (HWE), using *P* < 0.05 as the significance threshold. Adjusted odds ratios (AORs) and 95% confidence intervals (CIs) were used to measure associations between different genotypes and RPL, with a cutoff of *P* < 0.05 to indicate significant differences. Differences in hormone concentrations (estradiol (E2), FSH, LH, PRL, and TSH) between miRNA genotypes and alleles were evaluated using a one-way analysis of variance (ANOVA), with a post hoc Scheffé test, for all pairwise comparisons, and independent, two-sample Student’s *t*-tests, as appropriate. During ANOVA analysis, the Kruskal-Wallis test was used for small sample sizes and/or when the *P*-value of Levene’s test was less than 0.05. Data are presented as the mean ± standard deviation (SD). Statistical analyses were performed using GraphPad Prism v. 4.0 (GraphPad Software Inc., La Jolla, CA, USA) and StatsDirect v. 2.4.4 (StatsDirect Ltd., Altrincham, UK).

## 3. Results

In this study, we investigated the relationship between four miRNA polymorphisms, miR-25T>C, miR-32C>A, miR-125C>T, and miR-222G>T, and RPL, in a cohort of Korean women. Clinical profiles for RPL patients (n = 361) and control subjects (n = 272) are shown in Table 1. Controls and patients were matched by age. We found that measures of coagulation status, including PT, aPTT, and PLT counts, were increased in RPL patients relative to control subjects (*P* < 0.05). In addition, the levels of E2 and LH were significantly higher in RPL patients than in control subjects, whereas FSH levels were decreased in RPL subjects compared with those in control subjects. We then measured genotype frequencies for each miRNA polymorphism (Table 2) and found no statistically significant association between any single polymorphism and RPL (PL ≥ 2). In addition, we detected no significant differences in the frequencies of any of single polymorphism in subjects with more than three PLs or those with more than four PLs. Each group was confirmed to be in HWE (>0.05) (Table 2).

We then performed an allele combination analysis by measuring the association between different combinations of the four miRNA polymorphisms and RPL (Table 3 and Table S1). From this analysis, we detected a statistically significant association between the miR-25T/miR-32C/miR-125aT/miR-222T combination and the increased incidence of RPL (AOR, 4.361; 95% CI, 1.496–12.72; *P =* 0.003). When analyzing three-gene combinations, we found that the miR-32C/miR-125aT/miR-222T (AOR, 3.085; 95% CI, 1.254–7.588; *P =* 0.010) and the miR-25T/miR-125aT/miR-222T (AOR, 2.929; 95% CI, 1.183–7.257; *P =* 0.015) allele combinations were associated with the increased incidence of RPL. In addition, the miR-125aT/miR-222T allele combination (AOR, 2.417; 95% CI, 1.084–5.386; *P =* 0.026) was also associated with RPL incidence during our two-gene analyses. We then performed genotype combination analyses to test for possible genotype interactions (Table S2). However, we found no statistically significant differences in the odds ratios or *P*-values (<0.05) for any combination. Calculation of AORs, which are adjusted by the ages of the subjects, also revealed no statistically significant difference between the RPL and control groups. 

We next investigated whether we could detect any associations between any of the clinical parameters measured in our study and any of the miRNA genotypes examined, by one-way ANOVA analysis. In the control group (Table 4), the mean gestational age was significantly different between those with different miR-32C>A genotypes (*P* < 0.05). In addition, LH levels were increased in those with miR-25 TC and CC, and creatinine levels were decreased in the miR-222 GT genotype (*P* < 0.05). One-way ANOVA analysis in RPL patients (Table 5 and Table S3) revealed that PLT levels were significantly different between those with different miR-125a C>T genotypes, and blood urea nitrogen (BUN) levels decreased depending on the miR-25 allele (*P* < 0.05). Creatinine levels were also associated with the genotypes of the miR-25 and miR-32 alleles (*P* < 0.05). 

Lastly, we predicted targets for the miRNAs analyzed in this study using TargetScan (http://www.targetscan.org/vert_72) and miRBase (http://www.mirbase.org/). We found that miR-25 and miR-32 are predicted to bind the 3’UTR of fibrinogen, which plays a critical role in the coagulation pathway [2]. In addition, miR-125a-3p is predicted to bind to VEGF, which has been implicated in a number of diseases, including breast cancer and coronary artery disease, and has been associated with RPL [24,25]. miR-222 is predicted to bind KDR (also known as VEGFR2), another factor that is associated with RPL. Notably, as shown in our previous study, these four miRNAs also bind to and regulate TGF-β, a cytokine that functions during angiogenesis and female reproductive function [18].

## 4. Discussion

In recent years, it has become clear that miRNAs play essential roles in the regulation of a large number of cellular functions, including proliferation, apoptosis, steroidogenesis, and cell signaling. Individual miRNAs can also regulate multiple downstream target genes and, thus, mediate a wide array of effects. Our previous study found that miR-25, miR-32, miR-125, and miR-222 are involved in the regulation of the TGF-β signaling pathway [18]. Both miR-32 and miR-125a have been reported to induce apoptosis [26], and miR-32 functions to inhibit nuclear factor (NF)-κB, expression [27]. Notably, these miRNAs have also been implicated in female reproductive function. Specifically, miR-125a was linked to RPL and was shown to regulate the expression of leukemia inhibitory factor receptor (LIFR), which plays an important regulatory role at the embryo-endometrial interface [9]. In addition, the overexpression of miR-125a can induce abnormal pregnancies and the upregulation of miR-32 can lead to preeclampsia [19].

Our genotype analysis data (Table 2) showed no significant differences in RPL incidence for any single miRNA. However, abundant evidence supports the idea that miRNAs are associated with RPL [28,29]. In our study, E2 and LH levels were significantly higher in RPL patients compared with control subjects (Table 1; E2, *P =* 0.003; LH, *P* < 0.0001). Other studies have also reported significantly increased E2 and LH levels unexplained RPL patients. When E2 levels rise, FSH levels decline, which may cause a hormone imbalance, such as high basal estradiol levels and high FSH/LH ratios, which play a role in unexplained RPL [30]. Clinical factors associated with coagulation, such as aPTT, PT, and PLT, are also significantly different between control subjects and RPL patients, suggesting that thromboembolic diseases, such as thrombophilia, may be associated with RPL [31].

Mutations in TGF-β have been implicated in the pathogenesis of RPL [32], although this finding is somewhat controversial, as another study reported that TGF-β allele alterations are not associated with RPL occurrence in Caucasian Argentine populations. Notably, TGF-β can induce Th17 differentiation, which can be inhibited by interleukin (IL)-35, through the suppression of IL-17 production. IL-35 levels have been found to be significantly decreased in RPL patients compared with control subjects, and the IL-35/IL-17 ratio was also found to be significantly lower in RPL patients relative to control subjects [33]. These data suggested a link between TGF-β/Th17 signaling and RPL. Here, we found that combinations of polymorphisms in four miRNAs that regulate TGF-β signaling are associated with increased risk of RPL, further suggesting that this pathway is involved in RPL [18].

Many studies have revealed that miRNA polymorphisms can affect the levels of VEGF and VEGFRs, such as KDR, leading to the inhibition of the VEGF pathway and abnormal angiogenesis [6,34,35]. In particular, understanding the mechanisms underlying the miRNA-mediated regulation of the VEGF-VEGFR pathway during pregnancy and fetal development is critical, as angiogenesis plays key roles in establishing and maintaining pregnancy [31,36]. In rodents, the downregulation of VEGF and its receptors has been shown to cause fetal death, in utero [25], and alterations in KDR expression result in abnormal angiogenesis, both in vivo [31] and in vitro, and promote defects in fetal development, in vivo. In addition, a previous study found that the KDR Q472H polymorphisms are associated with RPL [37]. Here, our target prediction analysis found that these miRNAs are predicted to regulate the expression of vascular- and thrombosis-related genes. In particular, miR-25 and miR-32 are predicted to bind fibrinogen, which plays a critical role in the coagulation pathway [2], miR-125a-3p is predicted to regulate VEGF, and miR-222 is predicted to bind KDR. These results suggested that polymorphisms in these miRNAs may affect RPL, via the modulation of the blood coagulation status and angiogenesis-related pathways.

RPL is a multifactorial disease that is caused by many clinical factors and genetic factors. We have examined RPL from various angles, especially the genetic aspect directly and/or indirectly. In previous studies, we examined many microRNAs related with RPL and identified some of the microRNAs that are associated, such as miR-27a and miR-150. Functionally, microRNAs bind to each 3’UTR gene a regulate expression of mRNA. In our previous study, miR-150 binds SERPINE1 mRNA-binding protein 1 (SERBP1), which thus indirectly regulates the SERPINE1 (also known as PAI-1) level. PAI-1 affects thrombosis and fibrosis. Low concentrations of PAI-1 are important for maintaining pregnancy. As a result, miR-150 increases the risk of RPL [28]. Additionally, altered miR-27a increases the folate level, which is one of the necessary factors of pregnancy. As a result, miR-27a showed a protective factor for RPL [10]). Moreover, in this study, we focused on microRNA that are associated with VEGF, their receptor KDR and fibrinogen, and TGF-β. Apart from TGF-β, these genes are already well-known as angiogenesis, vasculogenesis, and thrombus, which are critical in maintaining pregnancy. In Table 1, we identify significantly different between RPL patients and controls in coagulation factors (e.g., PT, aPTT) for increasing PLT levels. It is supposed that the selected microRNAs regulate gene expression to affect thrombus. Based on our studies, we identified that many genes are related to RPL. However, we could not identify which of these was the most important or critical factor for RPL.

A number of miRNAs are currently used as biomarkers for disease diagnosis [38]. Here, our data suggested that the miRNAs investigated in this study could represent the potential biomarkers for the diagnosis of RPL [10,28]. These studies showed that these miRNAs, miR-25, miR-32, miR-125a, and miR-222, are likely to bind VEGF, KDR, fibrinogen, and TGF-β. Apart from TGF-β, the other potential binding targets are associated with angiogenesis, which is known to be associated with RPL [11,12]. The assessment of the combinations of linked genes may act as a better biomarker than the assessment of a single gene. To develop such a diagnostic tool, however, we will first need to determine how these miRNAs regulate mRNA expression and modulate cell signaling factors, both in vitro and in vivo. 

This study has several limitations. First, how the miRNA polymorphisms specifically affect RPL development remains unclear. In this study, we focused on SNPs found in four miRNAs and examined whether we could detect an association between the genotype frequencies of these polymorphisms and RPL incidence or the clinical parameters that may contribute to this condition. However, many factors contribute to successful pregnancies and fetal development, and the analysis of genotype frequencies is not sufficient to explain why implantation failure repeatedly occurs in some women. Second, our study population was limited to Korean women; therefore, it is necessary to confirm that our findings are generalizable to women from other ethnic groups. Third, miRNA polymorphisms may also affect miRNA expression, which, in turn, would affect their downstream genes; however, in this study, we did not measure the expression levels of each miRNA in patient tissues. Fourth, our patient sample size was small; therefore, additional studies, with larger sample sizes, are necessary to confirm our results.

## 5. Conclusion

We analyzed polymorphism frequencies in four microRNAs (miR-25, miR-32, miR-125a, and miR-222) between RPL patients and control subjects. The combination of miR-25C>T and miR-222G>T, two minor alleles, was associated with RPL occurrence. In addition, miRNAs polymorphisms appear to affect environmental factors. We suggest that interactions between miRNAs alleles and environmental factors may increase the risk of RPL.

## Figures and Tables

**Table 1:** Baseline clinical characteristics in recurrent pregnancy loss (RPL) patients and control subjects.

Characteristic	Control (*n* = 272)	RPL (*n* = 361)	*P*-Value
Age (years)	32.5 ± 4.02	32.5 ± 3.92	0.650
BMI (kg/m^2^)	21.6 ± 3.2	21.5 ± 3.92	0.754
Previous pregnancy losses (*n*)	N/A	3 ± 1.53	
Live births (*n*)	1.71 ± 0.58	N/A	
Mean gestational age (weeks)	39.1 ± 1.59	7.4 ± 1.94	
PT (sec)	11.2 ± 2.81	11.6 ± 0.87	**<0.0001**
aPTT (sec)	30.7 ± 4.65	32.3 ± 4.38	**0.006**
PLT (10^3^/μl)	238.3 ± 59.66	254.8 ± 55.85	**0.004**
Folate (mg/ml)	14 ± 8.52	14.3 ± 12.21	0.918
Total cholesterol (mg/dl)	239.5 ± 88.39	187.1 ± 49.34	**0.006**
Uric acid (mg/dl)	4.1 ± 1.5	3.8 ± 0.85	0.600
BUN (mg/dl)	8 ± 2.02	9.9 ± 2.69	**0.0001**
Creatinine (mg/dl)	0.7 ± 0.08	0.7 ± 0.12	0.076
E2 (Basal)	26 ± 14.75	35.4 ± 29.37	**0.003**
TSH (mU/l)	N/A	2.2 ± 1.57	
FSH (mU/l)	8.1 ± 2.85	7.4 ± 10.72	**<0.0001**
LH (mU/l)	3.3 ± 1.74	6.3 ± 12.38	**<0.0001**
Prolactin (ng/ml)	N/A	16 ± 13.22	
HDL (mg/dl)	N/A	61.6 ± 18.14	
Hct (%)	35.5 ± 4.22	37.4 ± 3.37	**<0.0001**
PAI-1 (ng/ml)	N/A	10.5 ± 5.74	
Hcy	N/A	6.9 ± 2.13	
TG (mg/dl)	N/A	179.1 ± 156.51	
CD56 (NK cells)	N/A	18.4 ± 8.04	

Data are presented as the mean ± standard deviation (SD). RPL, recurrent pregnancy loss; BMI, body mass index; PT, prothrombin time; aPTT, activated partial thromboplastin time; PLT, platelets; BUN, blood urea nitrogen; E2, estradiol; TSH, thyroid-stimulating hormone; FSH, follicle-stimulating hormone; LH, luteinizing hormone; HDL, high-density lipoprotein; Hct, hematocrit; PAI-1, plasminogen activator inhibitor-1; Hcy, homocysteine; TG, triglycerides; NK, natural killer. Significant *P*-values are marked in bold.

**Table 2:** Comparison of genotype frequencies for each miRNA polymorphism in RPL patients and control subjects.

Genotypes	Controls (*n =* 272)	PL ≥ 2 (*n =* 361)	AOR (95% CI) ^a^	*P*-Value	PL ≥ 3 (*n =* 190)	AOR (95% CI) ^a^	*P*-Value	PL ≥ 4 (*n =* 79)	AOR (95% CI) ^a^	*P*-Value
*miR-25* rs1527423 T>C										
TT	214 (78.7)	265 (73.4)	1.000 (reference)		139 (73.2)	1.000 (reference)		59 (74.7)	1.000 (reference)	
TC	53 (19.5)	87 (24.1)	1.326 (0.901–1.952)	0.153	47 (24.7)	1.377 (0.880–2.155)	0.162	19 (24.1)	1.300 (0.714–2.366)	0.390
CC	5 (1.8)	9 (2.5)	1.452 (0.479–4.402)	0.510	4 (2.1)	1.261 (0.331–4.811)	0.734	1 (1.3)	0.666 (0.320–1.387)	0.278
Dominant			1.339 (0.922–1.944)	0.125		1.368 (0.886–2.111)	0.157		0.780 (0.415–1.468)	0.442
Recessive			1.365 (0.452–4.119)	0.581		1.162 (0.307–4.397)	0.825		0.792 (0.458–1.371)	0.405
HWE	0.423	0.563			0.991			0.699		
*miR-32* rs7041716 C>A										
CC	221 (81.3)	291 (80.6)	1.000 (reference)		161 (84.7)	1.000 (reference)		69 (87.3)	1.000 (reference)	
CA	48 (17.6)	67 (18.6)	1.061 (0.704–1.598)	0.778	29 (15.3)	0.828 (0.500–1.372)	0.464	10 (12.7)	0.666 (0.320–1.387)	0.278
AA	3 (1.1)	3 (0.8)	0.753 (0.150–3.769)	0.730	0 (0)	N/A	0.994	0 (0)	N/A	0.995
Dominant			1.043 (0.698–1.558)	0.837		0.779 (0.473–1.285)	0.329		0.627 (0.302–1.301)	0.210
Recessive			0.749 (0.150–3.743)	0.725		N/A	0.994		N/A	0.995
HWE	0.828	0.689			0.255			0.548		
*miR-125a* rs12976445 C>T										
CC	203 (74.6)	263 (72.9)	1.000 (reference)		144 (75.8)	1.000 (reference)		62 (78.5)	1.000 (reference)	
CT	63 (23.2)	91 (25.2)	1.121 (0.773–1.624)	0.547	42 (22.1)	0.947 (0.606–1.479)	0.810	15 (19)	0.780 (0.415–1.468)	0.442
TT	6 (2.2)	7 (1.9)	0.910 (0.301–2.754)	0.868	4 (2.1)	0.922 (0.255–3.336)	0.902	2 (2.5)	1.098 (0.216–5.582)	0.911
Dominant			1.099 (0.767–1.575)	0.606		0.945 (0.614–1.453)	0.796		0.808 (0.442–1.476)	0.488
Recessive			0.878 (0.292–2.643)	0.817		0.935 (0.260–3.369)	0.919		1.163 (0.230–5.889)	0.856
HWE	0.672	789			0.652			0.362		
*miR-222* rs34678647 G>T										
GG	151 (55.5)	205 (56.8)	1.000 (reference)		110 (57.9)	1.000 (reference)		48 (60.8)	1.000 (reference)	
GT	103 (37.9)	140 (38.8)	1.007 (0.724–1.402)	0.967	68 (35.8)	0.925 (0.623–1.372)	0.697	25 (31.6)	0.792 (0.458–1.371)	0.405
TT	18 (6.6)	16 (4.4)	0.654 (0.323–1.324)	0.238	12 (6.3)	0.916 (0.424–1.98)	0.823	6 (7.6)	1.048 (0.392–2.797)	0.926
Dominant			0.951 (0.692–1.306)	0.756		0.920 (0.632–1.339)	0.661		0.823 (0.493–1.375)	0.457
Recessive			0.655 (0.327–1.308)	0.230		0.947 (0.444–2.018)	0.887		1.147 (0.439–3.002)	0.780
HWE	0.939	0.193			0.733			0.295		

RPL, recurrent pregnancy loss; PL, pregnancy losses; AOR, adjusted odds ratio; 95% CI, 95% confidence interval; N/A, not applicable, ^a^: Adjusted by age of participants.

**Table 3:** Allele combination analysis for miRNA polymorphisms in RPL patients and controls.

Allele Combinations	Overall (2*n* = 1266)	Controls (2*n* = 544)	RPL (2*n* = 722)	OR (95% CI)	*P*-Value
miR-25T>C/miR-32C>A/miR-125aC>T/miR-222G>T					
T-C-C-G	0.514	0.504	0.522	1.000 (reference)	
T-C-C-T	0.1641	0.1867	0.1473	0.755 (0.552–1.033)	0.079
T-C-T-G	0.0844	0.1035	0.0733	0.688 (0.458–1.033)	0.070
T-C-T-T	0.0239	0.0069	0.0328	4.361 (1.496–12.72)	**0.003**
T-A-T-T	0.0008	0.0032	0	0.145 (0.007–3.044)	0.178
C-C-T-G	0.0205	0.0122	0.0268	1.973 (0.818–4.759)	0.124
C-C-T-T	0.0012	0.0037	0	0.145 (0.007–3.044)	0.178
C-A-T-G	0.0024	0.0017	0.0006	0.242 (0.01–5.977)	0.241
miR-32C>A/miR-125aC>T/miR-222G>T					
C-C-G	0.5778	0.5591	0.5909	1.000 (reference)	
C-C-T	0.1923	0.2157	0.1748	0.767 (0.573–1.026)	0.074
C-T-G	0.1034	0.1142	0.0971	0.804 (0.554–1.166)	0.249
C-T-T	0.0262	0.0118	0.0361	3.085 (1.254–7.588)	**0.010**
miR-25T>C/miR-125aC>T/miR-222G>T					
T-C-G	0.5623	0.5524	0.5688	1.000 (reference)	
T-C-T	0.1868	0.2113	0.1698	0.783 (0.583–1.052)	0.104
T-T-G	0.0936	0.1089	0.0832	0.745 (0.505–1.099)	0.137
T-T-T	0.0246	0.0116	0.0327	2.929 (1.183–7.257)	**0.015**
C-C-G	0.0756	0.0686	0.0812	1.168 (0.754–1.808)	0.486
C-T-G	0.0228	0.0146	0.0285	1.922 (0.84–4.4)	0.116
C-T-T	0.0012	0.0028	0.001	0.366 (0.033–4.059)	0.577
miR-125aC>T/miR-222G>T					
C-G	0.6395	0.6211	0.6538	1.000 (reference)	
C-T	0.2183	0.241	0.2008	0.793 (0.602–1.043)	0.097
T-G	0.1149	0.1234	0.108	0.834 (0.584–1.189)	0.315
T-T	0.0273	0.0145	0.0374	2.417 (1.084–5.386)	**0.026**

RPL, recurrent pregnancy loss; OR, odds ratio; 95% CI, 95% confidence interval; N/A, not applicable. Significant *P*-values are marked in bold.

**Table 4:** Association between various clinical parameters and miRNA gene polymorphisms in control subjects.

Control															
Genotypes	Age (Years)	BMI (kg/m²)	Live Births (*n*)	Mean Gestational Age (Weeks)	PT (sec)	aPTT (sec)	PLT (10^3^/ul)	Folate (mg/ml)	Total Cholesterol (mg/dl)	Uric Acid (mg/dl)	BUN (mg/dl)	Creatinine (mg/dl)	E2 (Basal)	FSH (mU/l)	LH (mU/l)
	(272)	(143)	(99)	(122)	(59)	(99)	(216)	(18)	(14)	(9)	(40)	(40)	(111)	(111)	(108)
*miR-25*T>C															
TT	32.5 ± 4	21.5 ± 3.31	1.7 ± 0.58	39 ± 1.63	11.6 ± 3.24	30.7 ± 4.81	239.1 ± 59.64	15.1 ± 9.74	217.7 ± 75.63	3.8 ± 1.78	7.9 ± 1.99	0.7 ± 0.09	26.7 ± 15.07	8 ± 2.88	3.1 ± 1.53
TC	32.7 ± 4.15	22 ± 2.78	1.9 ± 0.54	39.5 ± 1.44	10.4 ± 0.92	30.5 ± 4.25	233.3 ± 59.77	11.7 ± 5.33	319.3 ± 100.05	4.8 ± 0.21	8.4 ± 2.25	0.6 ± 0.05	24 ± 14.38	8.5 ± 2.9	4 ± 2.17
CC	30.8 ± 3.96	20.1 ± 0.36	1.5 ± 0.71	40.3 ± 0.35	10.4	32.9	260.6 ± 65.53	-	-	-	-	-	22.2 ± 7.87	7.2 ± 1.26	5.6 ± 1.17
*P*	0.601	0.629	0.194	0.219	0.36	0.882	0.589	0.436	0.076	0.38	0.583	0.143	0.676	0.651	**0.006**
*miR-32*C>A															
CC	32.5 ± 4.03	21.5 ± 3.19	1.7 ± 0.55	39.3 ± 1.48	11.3 ± 2.92	30.8 ± 4.7	236.6 ± 61.56	14 ± 9.06	248.3 ± 98.3	4.3 ± 1.51	8.3 ± 2.04	0.7 ± 0.08	26.7 ± 14.92	8 ± 2.73	3.2 ± 1.66
CA	32.6 ± 4.09	22.5 ± 3.27	1.7 ± 0.7	38.2 ± 1.82	10.1 ± 1.6	29.6 ± 4.32	246 ± 49.85	13.8 ± 1.51	207.3 ± 22.01	3.5 ± 1.84	7 ± 1.67	0.6 ± 0.09	23.7 ± 14.07	8.8 ± 3.3	3.8 ± 2.07
AA	32.7 ± 4.04	20.9 ± 2.87	1	39.5 ± 0.71	11.4	35.7	250.5 ± 67.18	-	-	-	-	-	11.1	5.7	4.1
*P*	0.974	0.376	0.467	**0.010**	0.67	0.383	0.66	0.977	0.499	0.557	0.105	0.071	0.424	0.328	0.317
*miR-125a*C>T															
CC	32.5 ± 4.13	21.6 ± 3.09	1.7 ± 0.58	39.2 ± 1.66	11.4 ± 3.27	30.6 ± 4.33	234.5 ± 60.3	13.8 ± 9.13	247 ± 84.9	4.2 ± 1.65	7.8 ± 2.08	0.7 ± 0.09	26.1 ± 14.9	8 ± 2.43	3.3 ± 1.75
CT	32.4 ± 3.76	21.3 ± 3.52	1.6 ± 0.59	39.1 ± 1.35	10.8 ± 0.51	31.2 ± 6.15	247.1 ± 57.18	15 ± 5.62	212 ± 115.31	3.9 ± 1.2	9 ± 1.44	0.7 ± 0.04	25 ± 14.57	8.5 ± 3.83	3.4 ± 1.78
TT	33.3 ± 3.61	23.7 ± 4.04	1.8 ± 0.5	38.3 ± 0.87	10.4 ± 0.76	32.2 ± 0.67	276.5 ± 45.14	-	-	-	-	-	37.5 ± 12.09	8 ± 2.97	2 ± 0.42
*P*	0.85	0.391	0.735	0.524	0.711	0.674	0.127	0.836	0.564	0.809	0.213	0.984	0.517	0.696	0.548
*miR-222*G>T															
GG	32.7 ± 3.93	21.5 ± 3.19	1.7 ± 0.6	38.9 ± 1.39	11 ± 1.71	31.3 ± 5.3	239.7 ± 59.33	14.7 ± 11.09	195.7 ± 50.63	3.6 ± 1.21	8.4 ± 1.55	0.7 ± 0.06	25.6 ± 14.52	8 ± 2.38	3.4 ± 1.54
GT	32.1 ± 4.12	21.6 ± 3.27	1.7 ± 0.53	39.4 ± 1.58	10.9 ± 0.89	29.9 ± 3.34	234.4 ± 60.51	13.1 ± 4.35	272.8 ± 95.93	3.9 ± 1.24	7.5 ± 1.99	0.6 ± 0.09	27.3 ± 15.41	8.3 ± 3.51	3.2 ± 2.03
TT	33.6 ± 4.16	22.2 ± 3.17	2.2 ± 0.45	38.9 ± 2.93	16.5 ± 11.14	29.6 ± 3.27	248.2 ± 59.59	12.8	309.5 ± 143.54	6.9	7.5 ± 4.71	0.7 ± 0.12	22.6 ± 13.98	7.9 ± 2.55	3.3 ± 1.76
*P*	0.257	0.86	0.143	0.188	0.971	0.967	0.65	0.927	0.220	0.125	0.264	**0.048**	0.665	0.891	0.845

Data are presented as the mean ± standard deviation (SD). BMI; body mass index; PT, prothrombin time; aPTT, activated partial thromboplastin time; PLT, platelets; BUN, blood urea nitrogen; E2, estradiol. Significant *P*-values are marked in bold.

**Table 5:** Association between various clinical parameters and miRNA gene polymorphisms in RPL patients.

Patients													
Genotypes	Age (Years)	BMI (kg/m^2^)	Previous Pregnancy Losses (n)	Mean Gestational Age (Weeks)	aPTT (sec)	PLT (103/ul)	Folate (mg/ml)	Total Cholesterol (mg/dl)	Uric Acid (mg/dl)	BUN (mg/dl)	Creatinine (mg/dl)	Prolactin (ng/ml)	HDL (mg/dl)
	(361)	(332)	(361)	(174)	(200)	(194)	(207)	(170)	(167)	(188)	(187)	(197)	(17)
miR-25T>C													
TT	32.7 ± 3.81	21.5 ± 4.34	3 ± 1.6	7.6 ± 2.08	32.5 ± 4.48	253.9 ± 54.71	14.8 ± 13.49	189.9 ± 51.96	3.9 ± 0.89	10.1 ± 2.77	0.7 ± 0.12	15.7 ± 12.79	55.1 ± 15.14
TC	31.9 ± 4.1	21.4 ± 2.53	3 ± 1.35	6.7 ± 1.37	31.4 ± 4.1	259.8 ± 60.24	13 ± 8.06	177.7 ± 38.61	3.6 ± 0.66	9.2 ± 2.32	0.7 ± 0.12	17 ± 14.61	86.2 ± 14.06
CC	33.8 ± 5.04	21.8 ± 2.31	2.8 ± 1.3	7.5 ± 1.78	32.6 ± 3.77	238.6 ± 54.21	12.5 ± 6.16	201.6 ± 63.85	3.6 ± 1.07	8.1 ± 2.37	0.6 ± 0.16	10.1 ± 0.54	63.3 ± 4.24
P	0.123	0.921	0.826	0.051	0.278	0.668	0.635	0.304	0.160	**0.049**	**0.014**	0.681	0.017
miR-32C>A													
CC	32.6 ± 3.91	21.4 ± 4.22	3.1 ± 1.54	7.5 ± 2.07	32.5 ± 4.56	256 ± 55.54	15 ± 13.13	185.1 ± 48.11	3.7 ± 0.86	9.9 ± 2.82	0.7 ± 0.12	16.2 ± 13.99	60.9 ± 17.86
CA	32.1 ± 3.97	21.9 ± 2.22	2.7 ± 1.48	6.9 ± 1.33	31.4 ± 3.69	252.1 ± 58.27	11.4 ± 6.62	194.4 ± 54.75	4 ± 0.79	10 ± 1.99	0.7 ± 0.13	14.8 ± 9.36	66.3 ± 27.37
AA	34 ± 5	20.1 ± 3.39	2	9 ± 1.41	33.7 ± 1.63	223 ± 31.11	15.1 ± 8.61	185 ± 9.9	3.2 ± 1.41	6.3 ± 2.34	0.6 ± 0.06	N/A	N/A
P	0.534	0.554	0.102	0.118	0.283	0.668	0.258	0.597	0.215	0.065	**0.026**	0.555	0.711
miR-125aC>T													
CC	32.9 ± 3.81	21.4 ± 3.38	3.1 ± 1.60	7.4 ± 2.05	32 ± 4.48	254.4 ± 52.57	14 ± 11.98	187.4 ± 48.49	3.8 ± 0.85	9.6 ± 2.67	0.7 ± 0.12	14.9 ± 10.98	65.9 ± 19.09
CT	31.8 ± 4.02	21.8 ± 5.15	2.9 ± 1.36	7.2 ± 1.53	33.2 ± 3.84	251.3 ± 60.4	15.3 ± 13.31	184.6 ± 50.46	3.8 ± 0.75	10.6 ± 2.67	0.7 ± 0.11	19.3 ± 18.1	51.2 ± 11.06
TT	31 ± 5.51	20.1 ± 4.25	2.9 ± 0.90	5	24.9	376 ± 66.47	12.9 ± 3.79	215.3 ± 76.14	4.9 ± 2.76	8.4 ± 2.32	0.7 ± 0.26	11.8 ± 3.9	N/A
P	**0.040**	0.473	0.537	0.409	0.068	0.008	0.794	0.578	0.905	0.062	0.891	0.286	0.130
miR-222G>T													
GG	32.8 ± 4.14	21.6 ± 3.25	3 ± 1.47	7.5 ± 1.97	31.8 ± 4.54	258.8 ± 58.07	14 ± 8.3	184.3 ± 50.45	3.9 ± 0.91	9.5 ± 2.62	0.7 ± 0.13	15.9 ± 12.11	59.1 ± 23.2
GT	32.4 ± 3.64	21.4 ± 4.83	3 ± 1.6	7.3 ± 1.98	32.8 ± 4.05	250.6 ± 52.18	15 ± 16.52	188.6 ± 49.03	3.6 ± 0.78	10.3 ± 2.72	0.7 ± 0.12	16.4 ± 14.96	65.4 ± 16.46
TT	31.3 ± 3.36	20.9 ± 2.15	3.6 ± 1.63	6.9 ± 1.25	33.6 ± 4.93	240.5 ± 60.32	10.4 ± 5.2	203.7 ± 40.95	3.8 ± 0.46	9.9 ± 2.92	0.7 ± 0.11	11.8 ± 3.66	51.8 ± 6.86
P	0.254	0.807	0.315	0.667	0.246	0.471	0.666	0.506	0.054	0.143	0.178	0.709	0.610

Data are presented as the mean ± standard deviation (SD). BMI; body mass index; PT, prothrombin time; aPTT, activated partial thromboplastin time; PLT, platelets; BUN, blood urea nitrogen. Significant *P*-values are marked in bold.

## Supplementary Materials

The following are available online at https://www.mdpi.com/2073-4425/11/4/354/s1, Table S1: Allele combination analysis for miRNA polymorphisms in RPL patients and controls; Table S2: Genotype combination analysis for miRNA polymorphisms in RPL patients and controls; Table S3: Association between various clinical parameters and miRNA gene polymorphisms in RPL patients.
