# Correction: Kim, N.K., et al. Study of the Association between microRNA (miR-25T>C, miR-32C>A, miR-125C>T, and miR-222G>T) Polymorphisms and the Risk of Recurrent Pregnancy Loss in Korean Women. *Genes* 2020, *11*, 354

**DOI:** 10.3390/genes11080948

**Published:** 2020-08-17

**Authors:** Jeong Yong Lee, Jung Oh Kim, Han Sung Park, Chang Soo Ryu, Ji Hyang Kim, Young Ran Kim, Woo Sik Lee, Jung Ryeol Lee, Nam Keun Kim

**Affiliations:** 1 Department of Biomedical Science, College of Life Science, CHA University, Seongnam 13488, Korea; smilee3625@naver.com (J.Y.L.); jokim8505@gmail.com (J.O.K.); hahnsung@naver.com (H.S.P.); regis2040@nate.com (C.S.R.); 2 Department of Obstetrics and Gynecology, CHA Bundang Medical Center, School of Medicine, CHA University, Seongnam 13496, Korea; bin0902@chamc.co.kr (J.H.K.); happyimam@naver.com (Y.R.K.); 3 Department of Obstetrics and Gynecology, CHA Gangnam Medical Center, School of Medicine, CHA University, Seoul 06135, Korea; wooslee@cha.ac.kr; 4 Department of Obstetrics and Gynecology, Seoul National University Bundang Hospital, Seongnam 13620, Korea

The authors wish to make a correction to the published version of their paper [1].

In Section 2.2, the conditions described for genotype analysis were erroneously reported to be the same as the ones described in the article by Kim, N.K et al. [2] (this reference is cited as [18] in the original text). Consequently, for the corrected version, the authors have added the following information about the correct conditions used:

Samples were genotyped by polymerase chain reaction (PCR)-restriction fragment length polymorphism analysis utilizing the following primers and PCR conditions: the miR-25T>C polymorphism was detected using primers (forward) 5′-TGA CCC TTA AAG GGG TAG CTG-3′ and (reverse) 5′-CCA CCC TCT CAG TGA AGG TTA A-3′ the miR-32C>A polymorphism was detected using primers (forward) 5′-TCC AGT GTT GGG AGA GAT TTC TTC A-3′ and (reverse) 5′-CAG TGC CAG TGG GTG GAT-3′, the miR-125C>T polymorphism was detected using primers (forward) 5′-TGT CTC TGT GGC TTC TGT GT-3′ and (reverse) 5′-AGA GAC TGG CAA CAT GGT GT-3′ and the miR-222G>T polymorphism was detected using primers (forward) 5′-GGG CTC TGT GGA AGA AAA AAG AA-3′ and (reverse) 5′-AGC ACC TAA GAA AAT ATG TGG CC-3′, under PCR conditions of initial denaturation at 95 °C for 5 min; 35 cycles of denaturation at 95 °C for 30 s, annealing at 58 °C for 30 s, and extension at 72 °C for 30 s; and final extension at 72 °C for 5 min. Polymorphisms were detected by digesting the PCR products with the restriction endonucleases Hpy166II, BtsCI, Bsp1286I, and Sau96I, respectively. The endonucleases were purchased from New England Biolabs (Beverly, MA, USA)

The authors would like to apologize for any inconvenience caused. The change does not affect the scientific results. The published version will be updated on the article webpage, with a reference to this correction notice.
