# Supplementary material for: Study of the Association between microRNA (miR-25T>C, miR-32C>A, miR-125C>T, and miR-222G>T) Polymorphisms and the Risk of Recurrent Pregnancy Loss in Korean Women
Source: Genes (Basel). 2020 Mar 26;11(4):354. doi: 10.3390/genes11040354 (PMC7231175; doi:10.3390/genes11040354)
Supplement: Supplementary file 1 [file genes-11-00354-s001.pdf]

Supplementary Table 1. Allele combination analysis for miRNA polymorphisms in RPL patients and controls.

Allele combinations	Overall (2n=1266)	Controls (2n=544)	RPL (2n=722)	OR (95% CI)	Pa-value
miR-25T>C/miR-32C>A/miR-125aC>T/miR-222G>T					
T-C-C-G	0.514	0.504	0.522	1.000 (reference)	
T-C-C-T	0.1641	0.1867	0.1473	0.755 (0.552 - 1.033)	0.079
T-C-T-G	0.0844	0.1035	0.0733	0.688 (0.458 - 1.033)	0.070
T-C-T-T	0.0239	0.0069	0.0328	4.361 (1.496 - 12.72)	**0.003**
T-A-C-G	0.0491	0.0492	0.048	0.942 (0.557 - 1.594)	0.824
T-A-C-T	0.022	0.024	0.0218	0.895 (0.423 - 1.891)	0.770
T-A-T-G	0.009	0.0066	0.0095	1.272 (0.369 - 4.389)	0.768
T-A-T-T	0.0008	0.0032	0	0.145 (0.007 - 3.044)	0.178
C-C-C-G	0.0627	0.0547	0.0681	1.187 (0.734 - 1.919)	0.484
C-C-C-T	0.0289	0.0289	0.0287	0.954 (0.489 - 1.862)	0.890
C-C-T-G	0.0205	0.0122	0.0268	1.973 (0.818 - 4.759)	0.124
C-C-T-T	0.0012	0.0037	0	0.145 (0.007 - 3.044)	0.178
C-A-C-G	0.0123	0.0125	0.0135	1.038 (0.39 - 2.762)	0.940
C-A-C-T	0.0047	0.0021	0.0053	2.907 (0.323 - 26.17)	0.406
C-A-T-G	0.0024	0.0017	0.0006	0.242 (0.01 - 5.977)	0.241
C-A-T-T	0	0	0.0024	3.636 (0.174 - 76.09)	0.512
miR-32C>A/miR-125aC>T/miR-222G>T					
C-C-G	0.5778	0.5591	0.5909	1.000 (reference)	
C-C-T	0.1923	0.2157	0.1748	0.767 (0.573 - 1.026)	0.074
C-T-G	0.1034	0.1142	0.0971	0.804 (0.554 - 1.166)	0.249
C-T-T	0.0262	0.0118	0.0361	3.085 (1.254 - 7.588)	**0.010**
A-C-G	0.0618	0.0616	0.0624	0.942 (0.589 - 1.506)	0.804
A-C-T	0.026	0.0258	0.0264	0.966 (0.477 - 1.957)	0.924
A-T-G	0.0114	0.0096	0.0114	1.139 (0.369 - 3.517)	0.821
A-T-T	0.0012	0.0023	0.0009	0.712 (0.044 - 11.43)	1.000
miR-25T>C/miR-125aC>T/miR-222G>T					
T-C-G	0.5623	0.5524	0.5688	1.000 (reference)	
T-C-T	0.1868	0.2113	0.1698	0.783 (0.583 - 1.052)	0.104
T-T-G	0.0936	0.1089	0.0832	0.745 (0.505 - 1.099)	0.137
T-T-T	0.0246	0.0116	0.0327	2.929 (1.183 - 7.257)	**0.015**
C-C-G	0.0756	0.0686	0.0812	1.168 (0.754 - 1.808)	0.486
C-C-T	0.0331	0.0298	0.0347	1.144 (0.6 - 2.181)	0.682
C-T-G	0.0228	0.0146	0.0285	1.922 (0.84 - 4.4)	0.116
C-T-T	0.0012	0.0028	0.001	0.366 (0.033 - 4.059)	0.577
miR-25T>C/miR-32C>A/miR-222G>T					
T-C-G	0.5982	0.6078	0.593	1.000 (reference)	
T-C-T	0.1881	0.1926	0.1823	0.972 (0.725 - 1.304)	0.851
T-A-G	0.0583	0.056	0.059	1.108 (0.681 - 1.806)	0.679
T-A-T	0.0226	0.0277	0.0202	0.773 (0.373 - 1.605)	0.489
C-C-G	0.0834	0.0661	0.0949	1.482 (0.966 - 2.274)	0.070
C-C-T	0.0299	0.0343	0.0287	0.855 (0.452 - 1.616)	0.629
C-A-G	0.0144	0.0146	0.0148	1.063 (0.423 - 2.674)	0.896
C-A-T	0.0049	0.0009	0.0071	8.51 (0.469 - 154.6)	0.073
miR-25T>C/miR-32C>A/miR-125aC>T					
T-C-C	0.6785	0.6911	0.6684	1.000 (reference)	
T-C-T	0.1079	0.1099	0.1069	0.999 (0.695 - 1.437)	0.996
T-A-C	0.0713	0.0727	0.0706	0.993 (0.642 - 1.534)	0.973
T-A-T	0.0097	0.0105	0.0087	0.779 (0.249 - 2.434)	0.666
C-C-C	0.0911	0.0836	0.097	1.211 (0.813 - 1.803)	0.345
C-C-T	0.0223	0.0162	0.0266	1.643 (0.735 - 3.675)	0.222
C-A-C	0.017	0.0148	0.0185	1.265 (0.519 - 3.084)	0.604
C-A-T	0.0024	0.0013	0.0033	1.557 (0.141 - 17.25)	1.000
miR-125aC>T/miR-222G>T					
C-G	0.6395	0.6211	0.6538	1.000 (reference)	
C-T	0.2183	0.241	0.2008	0.793 (0.602 - 1.043)	0.097
T-G	0.1149	0.1234	0.108	0.834 (0.584 - 1.189)	0.315
T-T	0.0273	0.0145	0.0374	2.417 (1.084 - 5.386)	**0.026**
miR-32C>A/miR-222G>T					
C-G	0.6815	0.6729	0.6881	1.000 (reference)	
C-T	0.2182	0.2278	0.2108	0.903 (0.687 - 1.186)	0.462
A-G	0.0728	0.0716	0.0737	1.001 (0.648 - 1.546)	0.997
A-T	0.0275	0.0277	0.0275	0.982 (0.496 - 1.944)	0.958

miR-32C>A/miR-125aC>T					
C-C	0.7698	0.7751	0.7657	1.000 (reference)	
C-T	0.1298	0.1257	0.1332	1.077 (0.77 - 1.507)	0.663
A-C	0.088	0.0871	0.0889	1.039 (0.698 - 1.546)	0.850
A-T	0.0123	0.0122	0.0122	0.981 (0.362 - 2.657)	0.970
miR-25T>C/miR-222G>T					
T-G	0.6563	0.6613	0.6525	1.000 (reference)	
T-T	0.211	0.2229	0.202	0.922 (0.699 - 1.217)	0.567
C-G	0.0981	0.0832	0.1093	1.342 (0.908 - 1.984)	0.140
C-T	0.0346	0.0327	0.0362	1.104 (0.596 - 2.045)	0.753
miR-25T>C/miR-125aC>T					
T-C	0.7496	0.7635	0.739	1.000 (reference)	
T-T	0.1177	0.1207	0.1155	0.977 (0.69 - 1.384)	0.897
C-C	0.1082	0.0987	0.1155	1.217 (0.843 - 1.758)	0.295
C-T	0.0245	0.0172	0.0299	1.9 (0.865 - 4.17)	0.104
miR-25T>C/miR-32C>A					
T-C	0.7863	0.8008	0.7753	1.000 (reference)	
T-A	0.081	0.0834	0.0793	0.986 (0.654 - 1.487)	0.947
C-C	0.1134	0.0999	0.1236	1.283 (0.895 - 1.84)	0.174
C-A	0.0193	0.0159	0.0218	1.384 (0.606 - 3.163)	0.439

RPL, recurrent pregnancy loss; OR, odds ratio; 95% CI, 95% confidence interval; N/A, not applicable; aFisher's exact test.

Supplementary Table 2. Genotype combination analysis for miRNA polymorphisms in RPL patients and controls

Genotype combination		Control	RPL	COR (95% CI)	P-value	AOR (95% CI)a	Pa-value
SNP 1	SNP 2	(n=272)	(n=361)				
miR-25T>C	miR-32C>A						
TT	CC	70 (25.7)	216 (59.8)	1.000 (reference)		1.000 (reference)	
	CA	17 (6.3)	47 (13)	1.094 (0.677 - 1.77)	0.714	1.095 (0.677 - 1.771)	0.712
	AA	0 (0)	2 (0.6)	0.543 (0.09 - 3.287)	0.506	0.548 (0.090 - 3.318)	0.512
TC	CC	42 (15.4)	70 (19.4)	1.358 (0.882 - 2.09)	0.164	1.363 (0.885 - 2.098)	0.160
	CA	11 (4)	17 (4.7)	1.259 (0.575 - 2.758)	0.565	1.272 (0.580 - 2.788)	0.549
	AA	0 (0)	0 (0)	N/A	N/A	N/A	N/A
CC	CC	3 (1.1)	5 (1.4)	1.358 (0.32 - 5.761)	0.678	1.334 (0.314 - 5.671)	0.697
	CA	2 (0.7)	3 (0.8)	1.222 (0.202 - 7.396)	0.827	1.288 (0.212 - 7.838)	0.784
	AA	0 (0)	1 (0.3)	N/A	0.994	N/A	0.994
miR-25T>C	miR-125aC>T						
TT	CC	161 (59.2)	198 (54.8)	1.000 (reference)		1.000 (reference)	
	CT	47 (17.3)	62 (17.2)	1.073 (0.696 - 1.653)	0.751	1.087 (0.704 - 1.677)	0.707
	TT	6 (2.2)	5 (1.4)	0.678 (0.203 - 2.261)	0.527	0.667 (0.199 - 2.231)	0.511
TC	CC	39 (14.3)	60 (16.6)	1.251 (0.795 - 1.969)	0.333	1.259 (0.799 - 1.982)	0.321
	CT	14 (5.1)	25 (6.9)	1.452 (0.731 - 2.885)	0.287	1.498 (0.752 - 2.987)	0.251
	TT	0 (0)	2 (0.6)	N/A	0.995	N/A	0.995
CC	CC	3 (1.1)	5 (1.4)	1.355 (0.319 - 5.757)	0.680	1.367 (0.320 - 5.839)	0.673
	CT	2 (0.7)	4 (1.1)	1.626 (0.294 - 8.993)	0.577	1.630 (0.293 - 9.07)	0.577
	TT	0 (0)	0 (0)	N/A		N/A	N/A
miR-25T>C	miR-222G>T						
TT	GG	120 (44.1)	152 (42.1)	1.000 (reference)		1.000 (reference)	
	GT	81 (29.8)	101 (28)	0.984 (0.675 - 1.436)	0.935	1.002 (0.685 - 1.464)	0.993
	TT	13 (4.8)	12 (3.3)	0.729 (0.321 - 1.655)	0.450	0.736 (0.323 - 1.677)	0.466
TC	GG	28 (10.3)	47 (13)	1.325 (0.784 - 2.242)	0.294	1.337 (0.788 - 2.269)	0.281
	GT	20 (7.4)	37 (10.2)	1.461 (0.806 - 2.646)	0.212	1.477 (0.814 - 2.680)	0.199
	TT	5 (1.8)	3 (0.8)	0.474 (0.111 - 2.022)	0.313	0.461 (0.108 - 1.972)	0.296
CC	GG	3 (1.1)	6 (1.7)	1.579 (0.387 - 6.444)	0.524	1.545 (0.378 - 6.316)	0.545
	GT	2 (0.7)	2 (0.6)	0.79 (0.11 - 5.687)	0.815	0.878 (0.120 - 6.444)	0.898
	TT	0 (0)	1 (0.3)	N/A	0.994	N/A	0.994
miR-32C>A	miR-125aC>T						

CC	CC	164 (60.3)	213 (59)	1.000 (reference)		1.000 (reference)	
	CT	52 (19.1)	71 (19.7)	1.051 (0.697 - 1.587)	0.812	1.059 (0.701 - 1.599)	0.787
	TT	5 (1.8)	7 (1.9)	1.078 (0.336 - 3.458)	0.900	1.097 (0.341 - 3.526)	0.877
CA	CC	37 (13.6)	47 (13)	0.978 (0.607 - 1.575)	0.927	0.979 (0.608 - 1.577)	0.930
	CT	10 (3.7)	20 (5.5)	1.540 (0.702 - 3.379)	0.282	1.557 (0.707 - 3.429)	0.272
	TT	1 (0.4)	0 (0)	N/A	0.994	N/A	0.994
AA	CC	2 (0.7)	3 (0.8)	1.155 (0.191 - 6.992)	0.875	1.148 (0.189 - 6.963)	0.881
	CT	1 (0.4)	0 (0)	N/A	0.994	N/A	0.994
	TT	0 (0)	0 (0)	N/A	N/A	N/A	N/A
miR-32C>A	miR-222G>T						
CC	GG	123 (45.2)	167 (46.3)	1.000 (reference)		1.000 (reference)	
	GT	86 (31.6)	113 (31.3)	0.968 (0.672 - 1.394)	0.860	0.976 (0.678 - 1.407)	0.898
	TT	12 (4.4)	11 (3)	0.675 (0.288 - 1.581)	0.365	0.673 (0.287 - 1.576)	0.362
CA	GG	25 (9.2)	35 (9.7)	1.031 (0.587 - 1.812)	0.915	1.032 (0.588 - 1.814)	0.912
	GT	17 (6.3)	27 (7.5)	1.170 (0.611 - 2.241)	0.636	1.170 (0.610 - 2.245)	0.637
	TT	6 (2.2)	5 (1.4)	0.614 (0.183 - 2.057)	0.429	0.613 (0.183 - 2.056)	0.428
AA	GG	3 (1.1)	3 (0.8)	0.737 (0.146 - 3.711)	0.711	0.732 (0.145 - 3.692)	0.706
	GT	0 (0)	0 (0)	N/A	N/A	N/A	N/A
	TT	0 (0)	0 (0)	N/A	N/A	N/A	N/A
miR-125aC>T	miR-222G>T						
CC	GG	160 (58.8)	150 (41.6)	1.000 (reference)		1.000 (reference)	
	GT	80 (29.4)	102 (28.3)	0.901 (0.614 - 1.323)	0.595	0.914 (0.621 - 1.343)	0.646
	TT	17 (6.3)	11 (3)	0.457 (0.206 - 1.016)	0.055	0.458 (0.206 - 1.017)	0.055
CT	GG	41 (15.1)	52 (14.4)	0.896 (0.555 - 1.447)	0.654	0.901 (0.558 - 1.456)	0.671
	GT	21 (7.7)	34 (9.4)	1.144 (0.629 - 2.081)	0.659	1.156 (0.631 - 2.117)	0.639
	TT	1 (0.4)	5 (1.4)	3.533 (0.407 - 30.682)	0.252	3.627 (0.417 - 31.557)	0.243
TT	GG	4 (1.5)	3 (0.8)	0.530 (0.116 - 2.417)	0.412	0.543 (0.118 - 2.498)	0.433
	GT	2 (0.7)	4 (1.1)	1.413 (0.254 - 7.858)	0.693	1.369 (0.245 - 7.635)	0.721
	TT	0 (0)	0 (0)	N/A	N/A	N/A	N/A

RPL, recurrent pregnancy loss; SNP, single nucleotide polymorphism; COR, crude odds ratio; AOR, adjusted odds ratio; 95% CI, 95% confidence interval; N/A, not applicable.

a Adjusted by age of participants.

Supplementary Table 3. Association between various clinical parameters and miRNA gene polymorphisms in RPL patients.

Patients											
Genotypes	Age (years) (361)	BMI (kg/m²) (332)	Previous pregnancy losses (n) (361)	Mean gestational age (weeks) (174)	PT (sec) (59)	aPTT (sec) (200)	PLT (103/ul) (194)	Folate (mg/ml) (207)	Total cholesterol (mg/dl) (170)	Uric acid (mg/dl) (167)	BUN (mg/dl) (188)
miR-25T>C											
TT	32.7±3.81	21.5±4.34	3±1.6	7.6±2.08	11.6±0.85	32.5±4.48	253.9±54.71	14.8±13.49	189.9±51.96	3.9±0.89	10.1±2.77
TC	31.9±4.1	21.4±2.53	3±1.35	6.7±1.37	11.5±0.93	31.4±4.1	259.8±60.24	13±8.06	177.7±38.61	3.6±0.66	9.2±2.32
CC	33.8±5.04	21.8±2.31	2.8±1.3	7.5±1.78	11.3±0.82	32.6±3.77	238.6±54.21	12.5±6.16	201.6±63.85	3.6±1.07	8.1±2.37
P	0.123	0.921	0.826	0.051	0.469	0.278	0.668	0.635	0.304	0.160	**0.049**
miR-32C>A											
CC	32.6±3.91	21.4±4.22	3.1±1.54	7.5±2.07	11.6±0.88	32.5±4.56	256±55.54	15±13.13	185.1±48.11	3.7±0.86	9.9±2.82
CA	32.1±3.97	21.9±2.22	2.7±1.48	6.9±1.33	11.4±0.85	31.4±3.69	252.1±58.27	11.4±6.62	194.4±54.75	4±0.79	10±1.99
AA	34±5	20.1±3.39	2	9±1.41	11.8±0.21	33.7±1.63	223±31.11	15.1±8.61	185±9.9	3.2±1.41	6.3±2.34
P	0.534	0.554	0.102	0.118	0.411	0.283	0.668	0.258	0.597	0.215	0.065
miR-125aC>T											
CC	32.9±3.81	21.4±3.38	3.1±1.60	7.4±2.05	11.6±0.82	32±4.48	254.4±52.57	14±11.98	187.4±48.49	3.8±0.85	9.6±2.67
CT	31.8±4.02	21.8±5.15	2.9±1.36	7.2±1.53	11.6±1.02	33.2±3.84	251.3±60.4	15.3±13.31	184.6±50.46	3.8±0.75	10.6±2.67
TT	31±5.51	20.1±4.25	2.9±0.90	5	10.6	24.9	376±66.47	12.9±3.79	215.3±76.14	4.9±2.76	8.4±2.32
P	**0.04**	0.473	0.537	0.409	0.498	0.068	**0.008**	0.794	0.578	0.905	0.062
miR-222G>T											
GG	32.8±4.14	21.6±3.25	3±1.47	7.5±1.97	11.6±0.87	31.8±4.54	258.8±58.07	14±8.3	184.3±50.45	3.9±0.91	9.5±2.62
GT	32.4±3.64	21.4±4.83	3±1.6	7.3±1.98	11.5±0.85	32.8±4.05	250.6±52.18	15±16.52	188.6±49.03	3.6±0.78	10.3±2.72
TT	31.3±3.36	20.9±2.15	3.6±1.63	6.9±1.25	11.9±1.05	33.6±4.93	240.5±60.32	10.4±5.2	203.7±40.95	3.8±0.46	9.9±2.92
P	0.254	0.807	0.315	0.667	0.522	0.246	0.471	0.666	0.506	0.054	0.143

Data are presented as the mean ± standard deviation (SD). BMI; body mass index; PT, prothrombin time; aPTT, activated partial thromboplastin time; PLT, platelets; BUN, blood urea nitrogen.

Supplementary Table 3. (continued)

Genotypes	Creatinine (mg/dl) (187)	E2 (Basal) (158)	TSH (mU/l) (201)	FSH (mU/l) (187)	LH (mU/l) (188)	Prolactin (ng/ml) (197)	HDL (mg/dl) (17)	Hct (%) (193)	Hcy (264)
miR-25T>C									
TT	0.7±0.12	37±32.03	2.3±1.6	7.9±12.28	6.6±14.23	15.7±12.79	55.1±15.14	37.4±3.34	6.9±2.16
TC	0.7±0.12	30.7±19.32	2.1±1.52	6±3.86	5.7±4.75	17±14.61	86.2±14.06	37.5±3.5	7.1±2.09
CC	0.6±0.16	34.1±21.57	1.4±0.29	7.4±2.87	6.4±4.31	10.1±0.54	63.3±4.24	35.8±3.4	5.9±1.17
P	**0.014**	0.517	0.539	0.577	0.905	0.681	**0.017**	0.562	0.351
miR-32C>A									
CC	0.7±0.12	32.8±23.98	2.3±1.6	7.7±11.83	6.7±13.72	16.2±13.99	60.9±17.86	37.3±3.52	7±2.2
CA	0.7±0.13	48.1±45.66	2±1.42	6±2.77	4.8±3	14.8±9.36	66.3±27.37	37.5±2.92	6.7±1.77
AA	0.6±0.06	27.9	N/A	5.7	4.6	N/A	N/A	37.9±1.06	6±2.27
P	**0.026**	0.139	0.349	0.689	0.695	0.555	0.711	0.95	0.458
miR-125aC>T									
CC	0.7±0.12	34.5±31.45	2.1±1.41	8.1±12.45	6.8±14.43	14.9±10.98	65.9±19.09	37.2±3.43	7±2.12
CT	0.7±0.11	36.7±22.34	2.4±2	5.6±2.69	4.9±3.26	19.3±18.1	51.2±11.06	37.8±3.16	6.7±2.19
TT	0.7±0.26	55.1±14.38	2.4±0.52	5.5±2.27	7.5±6.47	11.8±3.9	N/A	36.6±5.37	6.2±1.9
P	0.891	0.469	0.539	0.368	0.656	0.286	0.130	0.509	0.413
miR-222G>T									
GG	0.7±0.13	35.3±26.83	2.2±1.6	7.5±11.7	5.7±7.22	15.9±12.11	59.1±23.2	37.6±3.47	6.8±2.23
GT	0.7±0.12	32.4±20.82	2.2±1.58	7.4±9.68	7.3±17.31	16.4±14.96	65.4±16.46	37.1±3.21	7±1.9
TT	0.7±0.11	79.1±94.92	2.3±0.55	5.2±2.08	3.8±2.29	11.8±3.66	51.8±6.86	36.7±3.75	7.5±2.77
P	0.178	0.414	0.972	0.896	0.622	0.709	0.610	0.551	0.524

Data are presented as the mean ± standard deviation (SD). E2, estradiol; TSH, thyroid-stimulating hormone; FSH, follicle-stimulating hormone; LH, luteinizing hormone; HDL, high-density lipoprotein; Hct, hematocrit; Hcy, homocysteine;
